# Nasal Mucoadhesive *in situ* Gel of Ondansetron Hydrochloride

**Published:** 2009

**Authors:** A. V. Bhalerao, S. L. Lonkar, S. S. Deshkar, S. V. Shirolkar, A. D. Deshpande

**Affiliations:** Padm. Dr. D. Y. Patil Institute of Pharmaceutical Sciences and Research, Pimpri, Pune-411 018, India

**Keywords:** Ondansetron hydrochloride, *in situ* Gel, intranasal delivery system, bioadhesive polymer

## Abstract

Ondansetron is a serotonin receptor antagonist used in the management of nausea and vomiting that is associated with cancer chemotherapy. There is a need for intranasal delivery due to poor bioavailability of drug because of first pass effect. The objective of this study was to develop an intranasal delivery system of ondansetron hydrochloride using thermo-sensitive polymer PF127 and mucoadhesive polymer hydroxypropylcellulose. Due to increase in bioadhesive polymer concentration, there was increase in bioadhesion strength, at the same time there was decrease in the spredability. An *in vitro* diffusion study revealed that viscosity of the vehicle has an influence on drug. The release of ondansetron hydrochloride from the gel matrix showed diffusion- controlled.

Ondansetron hydrochloride is a serotonin (5HT_3_) receptor antagonist used in the management of nausea and vomiting that is associated with cancer chemotherapy. There is a need for intranasal delivery due to poor bioavailability of drug because of first pass effect[1]. The objective of this study was to develop an intranasal delivery system of ondansetron hydrochloride using thermo sensitive polymer PF127 and mucoadhesive polymer.

## MATERIALS AND METHODS

Lutrol F127 (PF127 and hydroxypropylcellulose (Klucel LF) was procured from Signet Chemicals Mumbai. Ondansetron hydrochloride was received as a gift sample from Dr. Reddy's Lab Hyderabad.

Nasal formulations consisting of aqueous gels of PF127 containing 18% (w/v) of polymer were prepared using the method described by Schmolka[2]. Composition was given in Table 1. The prepared formulations were evaluated for appearance, clarity, pH, gelation temperature, spredability, bioadhesion strength, viscosity, drug content and diffusion study. Gelation temperature (TG) was measured by visual inspection using a modification of Miller and Donavan technique[3]. Rheological studies were performed using a Brookfield digital CAP 2000+viscometer to determine viscosity. Spreadability in terms of flow ability of various mucoadhesive thermoreversible gels was determined[4]. The bioadhesive potential of each formulation was determined by measuring the weight required to detach the formulation from nasal mucosal tissue using a method described by Yong *et al.*[5].

**TABLE 1:** THE FORMULAE FOR THE PREPARATION OF IN SITU NASAL GELS WITH VARYING CONCENTRATION OF HYDROXYLPROPYLCELLULOSE

Ingredient	F1	F2	F3	F4	F5
Ondansetron hydrochloride (mg)	50	50	50	50	50
Poloxamer 407 (mg)	900	900	900	900	900
Propylene glycol (ml)	0.9	0.9	0.9	0.9	0.9
Transcutol-P (ml)	0.1	0.1	0.1	0.1	0.1
Sodium metabisulphite (mg)	12.5	12.5	12.5	12.5	12.5
Benzalkonium chloride (mg)	1	1	1	1	1
Hydroxypropylcellulose (%)	-	0.2	0.3	0.5	0.7
Distilled water q.s.	5ml	5ml	5ml	5ml	5ml

### *In vitro* release studies were carried out in the following manner:

A glass cup with a cross-sectional area of 7.5 cmnd and adhesive tape, and inverted under the surface of 500 ml of simulated nasal fluid of pH 5.5 at 35±0.5° in USP XXIII Type I Dissolution Test Apparatus with a speed of 50 rpm. Five millilitres of aliquots were withdrawn at specified time intervals and immediately replaced with fresh dissolution medium[6]. The drug content in the withdrawn samples was determined spectrophotometrically at 310 nm using a UV/Vis spectrophotometer and simulated nasal electrolyte solution as a blank.

## RESULTS AND DISCUSSION

**Fig. 1:**
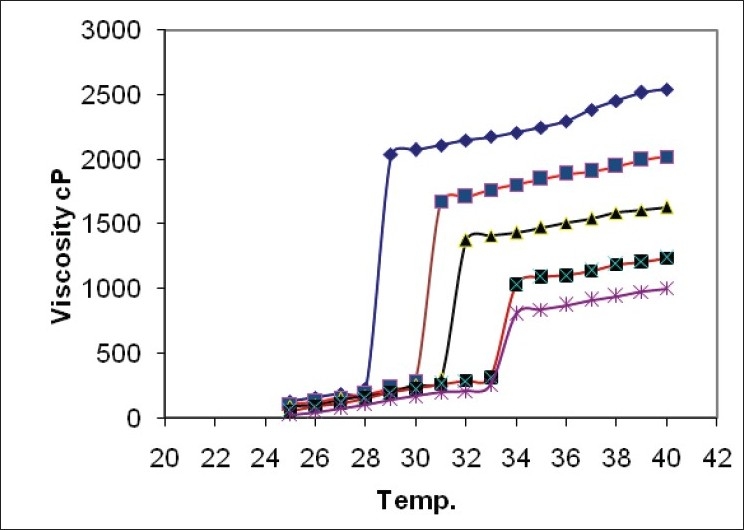
Viscosity of gels containing various concentrations of hydroxylpropylcellulose Viscosity measurements at different temperatiures were made for gels containing various concentrations of HPC (hydroxypropylcellulose), (–◆–) F5 0.7% HPC, (–■–)F4 0.5% HPC, (–▲–) F3 0.3% HPC, (–■–) F2 0.2% HPC and (–*–) F1 plain.

**Fig. 2:**
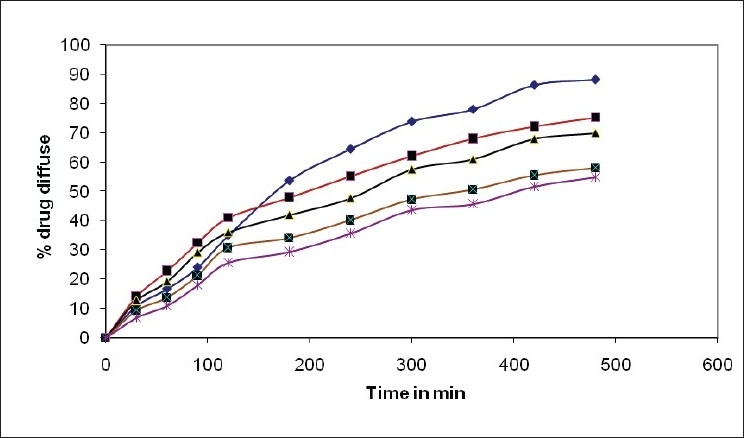
Diffusion profiles of ondansetron hydrochloride from *in situ* nasal gels Diffusion profiles of ondansetron hydrochloride from in situ nasal gels containing various concentrations of bioadhesive polymer hydroxypropylcellulose, (–◆–) F1, (–■–) F2, (–▲–) F3, (–■–) F4 and (–*–) F5.

All gels were glassy clear in appearance. Gel pH was in the range of 5.3 to 5.6 which was in the range of pH at the absorption site (4.5-6.5). In the preliminary studies, the minimum concentration of PF127 that formed gel below 34° was found to be 18% wt/vol. The plain formulation (F1) without bioadhesive polymer has minimum bioadhesion 529.2 dyne/cm^2^ but maximum spredability and 88.2% drug release after 8 h. In all formulations 0.2% w/v bioadhesive polymer showed maximum spredability while 0.7% w/v showed minimum spredability (Table 2). There was drastic increase in viscosity at gelation temperature (Table 3, fig. 1). It was observed that the concentration of bioadhesive polymer increased from 0.2% to 0.7% showed retardation of ondansetron hydrochloride release from 75.2% to 54.9% (fig. 2).

**TABLE 2:** CHARACTERISTICS OF ALL FORMULATIONS

Formulation	Appearance	pH	Gelation Temperature (°)	Drug content (%)	Bioadhesive Strength (dyne/cm^2^)	Spredability in distance (cm)
F1	+++	5.3	34±1.15	99.81±0.08	529.2	8.4
F2	+++	5.4	33±0.57	97.16±1.7	1862	7.1
F3	+++	5.4	32±0.57	98.96±0.89	2018.8	5.9
F4	+++	5.4	31±0.57	100.51±0.63	2381.4	5.3
F5	+++	5.6	29±0.57	100.51±0.05	2508.8	4.3

Appearance, pH, gelation temperature, drug content, bioadhesive strength and spredability of all formulations.

+++ represents glassy clear, n=3

**TABLE 3:** VISCOSITY OF NASAL FORMULATION AT DIFFERENT TEMPERATURES

Formulation	Viscosity at 25° in cP	Viscosity at 35° in cP	Gelling point by graph (°)
F1	25	839	34
F2	51	1089	34
F3	88	1474	32
F4	107	1847	31
F5	129	2247	29

Viscosity by Brookfield viscometer cap 2000+ spindle No. 1 at 5 rpm of nasal formulation at different temperature 250 for solutions and 350 forgels

Various solubilizers were tried during preformulation study. Propylene glycol and Transcutol were selected as solubilizers for further study. Poloxamer 407 gives gelation temperature in nasal temperature range 28° to 35° in 18% w/v concentration. Addition of bioadhesive polymer from 0.2% to 0.7% further lowered the gelation temperature from 34° to 29°. Gelation temperatures obtained using two different methods (visual inspection and rheological method) did not vary more than ±1°. The gelation temperature lowering effect of bioadhesive polymer might have caused in part by the increased viscosity after dissolution of mucosadhesive polymer. The pH values of all formulations were found in the range of 5.3 to 5.6. Formulation should posses' mild acidic pH for activation of lysozyme (A natural antibacterial enzyme important for controlling nasal microbial count which becomes inactive at alkaline pH). The pKa of ondansetron hydrochloride is 7.4; so drug present in solubilised form in pH range 5 to 6.5 (ondansetron hydrochloride precipitate above 6.8). It was observed that the concentration of bioadhesive polymer increased from 0.2% to 0.7% showed retardation of ondansetron hydrochloride release, probable mechanism for such retardation of release may be reduction in number and dimensions of the channels in gel structure by increased viscosity of the formulation. From the study it can be concluded that the nasal *in situ* gels can be formulated of ondansetron hydrochloride using PF127 and bioadhesive polymer.
